# Epirubicin and docetaxel as neoadjuvant treatment of hormone receptor positive, HER-2 negative breast cancer: findings from two successive phase II studies

**DOI:** 10.2478/raon-2013-0012

**Published:** 2013-02-01

**Authors:** Alessandro Tuzi, Davide Lombardi, Diana Crivellari, Loredana Militello, Tiziana Perin, Manuela La Grassa, Samuele Massarut, Andrea Veronesi

**Affiliations:** 1 Divisions of Medical Oncology C, Centro di Riferimento Oncologico, Via Franco Gallini 2, 33081 Aviano, Italy.; 2 Pathology, Centro di Riferimento Oncologico, Via Franco Gallini 2, 33081 Aviano, Italy.; 3 Radiology, Centro di Riferimento Oncologico, Via Franco Gallini 2, 33081 Aviano, Italy.; 4 Breast Surgery; Centro di Riferimento Oncologico, Via Franco Gallini 2, 33081 Aviano, Italy.

**Keywords:** breast cancer, docetaxel, epirubicin, neoadjuvant chemotherapy

## Abstract

**Background:**

We report on the activity of the combination of epirubicin and docetaxel given in neoadjuvant setting for 4 and 8 cycles respectively in 2 successive series of patients with large operable or locally advanced, hormone receptor positive, HER-2 negative breast cancer.

**Patients and methods:**

Patients were treated from 2002 to 2006 with epirubicin 90 mg/m^2^ and docetaxel 75 mg/m^2^ intravenously, every 3 weeks for 4 cycles before and 4 cycles after surgery (Series I – 13 patients), and from 2006 to 2010 with the same regimen administered for 8 cycles preoperatively (Series II – 37 patients), plus hormonal therapy for 5 years and radiation therapy if indicated. All Series I and 32 Series II patients were able to complete the preoperative chemotherapy.

**Results:**

A complete response was found in 1 patient from Series I and 13 patients from Series II and the partial remission in 10 patients from Series I and 21 patients from Series II. Two Series I and 3 Series II patients did not respond clinically. Response rate (Series I/Series II) was 84/92%. All 50 patients underwent surgery. In Series I patients, 3 pCR occurred in the breast and the axilla was histologically negative in 2 cases. No evidence of disease both in the breast and in the axilla was achieved in 7.6% (1/13) of patients. In Series II patients, 8 pCR occurred in the breast and axilla was histologically negative in 15 patients. No evidence of disease both in the breast and in the axilla occurred in 10.8% (4/37) of patients. G3–G4 toxicity included myelosuppression in 3 patients from Series I and all patients from Series II, and mucositis in 1 patient from Series I and 4 patients from series II. No other G3–4 toxicities or toxic deaths occurred. Five-year progression free survival was 38% and 90% in Series I and Series II patients respectively.

**Conclusions:**

The incidence of pathologic complete remissions was lower in our patient population, compared to reported data. A longer duration of the preoperative treatment might be associated with a longer progression-free survival.

## Introduction

The identification of subgroups of breast cancers with different behaviour and different response to therapy has profoundly modified the approach to breast cancer, both in the adjuvant^1^ and in the advanced settings.

Preoperative or neoadjuvant chemotherapy is a standard of care for cases of breast cancer not amenable to conservative surgery.^2^,^3^ The achievement of a pathological complete response (pCR) has a prognostic value, regardless of the hormone receptor status of the tumour.^4^ Advantages of such approach include the possibility to perform smaller resections with better cosmetic outcome^5^,^6^ and an early assessment of response to chemotherapy.^3^,^7^ The pathological complete response rate is in the order of 20–25%, with a considerable degree of interstudy variability.^3^,^8^

There is an agreement on the use of the two most active classes of drugs (anthracyclines and taxanes) in the neoadjuvant setting. The combination of epirubicin and docetaxel (ET) is widely accepted in this situation.^9^,^10^

Patients with hormone receptor positive, HER-2 negative disease, defined as Luminal A and B cases^11^, have been found to respond less well to neoadjuvant chemotherapy, particularly in terms of achieving pCR compared to patients with HER-2 positive or triple negative disease.^12^–^14^ For example, in a large pooled analysis of 7 neoadjuvant trials, pCR rates were 9% in Luminal A and B cases, 32% in HER-2 positive cases and 34% in triple negative cases.

In March 2002, we started a study that evaluated the activity of four cycles of neoadjuvant ET in patients with breast cancer. Patients were not selected according to the molecular subtype. The results of that study have been reported previously.^15^ Since 2006, the neoadjuvant treatment was changed and for the subsequent study hormone receptor positive, HER-2 negative patients were selected only. They were treated with ET at the same dosages as in the previous study, but received 8 instead of 4 neoadjuvant chemotherapy cycles.

In this paper we descriptively present the updated outcome of the group of hormone receptor positive, HER-2 negative cases (selected cases from previous study) treated with 4 cycles of neoadjuvant ET (Series I) and the outcome of patients treated with 8 cycles of neoadjuvant ET that were included in the subsequent study (Series II). No formal comparison was made throughout.

## Patients and methods

Eligibility criteria for Series I and II were the same and included: histologically confirmed invasive breast cancer, oestrogen (ER) and/or progesterone receptor (PR) positive, HER-2 negative, operable T2–T4 disease, unsuitable for conservative surgery, no evidence of distant disease, no previous antineoplastic treatment, adequate bone marrow (absolute neutrophil count ^3^2.0 x 10^9^/l, platelet count ^3^100 x 10^9^/l), renal, liver and cardiac (left ventricular ejection fraction ^3^50% by echocardiography) functions. Pre-treatment evaluation included mammography and breast ultrasound, routine blood tests, chest X-ray, abdominal CT scan or ultrasound, electrocardiogram and echocardiogram. Bone scan was performed in the presence of symptoms. All patients provided written informed consent prior to the initiation of treatment. ER, PR, HER2 and Ki-67 status was evaluated by immunohistochemistry (IHC). IHC analysis was performed on formalin-fixed, paraffin-embedded breast cancer tissue using specific primary rabbit monoclonal antibodies to ER (SP1, Ventana, ready to use), PgR (1E2, Ventana, ready to use), ki-67 (30–9, Ventana, ready to use) and HER2 (4B5, Ventana, ready to use) with an autostaining system (Ventana Medical System, Tucson, Arizona). ER, PR and Ki-67 immunostaining results were recorded as the percentage of immunoreactive cells over at least 2000 tumor cells randomly selected from the periphery of invasive carcinoma in surgical specimens. HER2 positivity was defined as 3+ overexpression by IHC and/or as 2.2 or greater HER2-toCEP17 ratio by SISH according to American Society Oncology/College of American Pathologist Guidelines.

Chemotherapy consisted of epirubicin 90 mg/m^2^ and docetaxel 75 mg/m^2^ administered intravenously every 3 weeks for 4 cycles preoperatively in Series I and for 8 cycles in Series II. Prednisone 25 mg orally was administered every 6 hours 3 times before and 3 times after chemotherapy. 5-HT3 based antiemetic treatment was provided. Complete blood counts were prescribed weekly. Prophylactic oral antibiotics were recommended in cases with neutrophil count below 0.5 x 10^9^/l. Therapy was administered every three weeks, provided that neutrophil count was >2.0 x 10^9^/l and the platelet count was >100 x 10^9^/1 on the day scheduled for the retreatment. G-CSF support (30 MU/day subcutaneously from day 2–11) was initiated in individual patients at the subsequent cycle in cases with febrile neutropenia or failure of neutrophil count recovery by the day of the retreatment. The epirubicin dose was decreased to 75 mg/m^2^ in instances of grade 4 thrombocytopenia, grade >3 non-haematological toxicity (except for alopecia, nausea/vomiting, musculoskeletal pain), persistence of grade ^3^2 non-hematologic toxicity at scheduled retreatment or febrile neutropenia despite G-CSF support. Treatment was discontinued in instances of congestive heart failure of any grade and/or of a significant reduction in left ventricular ejection fraction (≥10 % decrease from baseline associated with a decline to a level <50 %) confirmed by an echocardiogram performed at one week interval.

Patients in Series II underwent mammography after 4 cycles to rule out the disease progression. At completion of preoperative chemotherapy (4 cycles in Series I, 8 cycles in Series II), repeat mammography and breast ultrasound were performed. Modified radical mastectomy or breast sparing surgery was performed. After surgery, 4 additional chemotherapy cycles as above were administered to Series I patients in the event of clinical response to pre-surgery chemotherapy. Patients in Series II received postoperatively Cyclophosphamide, Methotrexate and Fluorouracil (classical CMF) for 3 cycles. At completion of chemotherapy, hormonal therapy (tamoxifen plus LHRH analogue in premenopausal, aromatase inhibitor in postmenopausal patients) was prescribed to all patients. Radiation therapy was indicated after chemotherapy according to institutional guidelines. In the event of breast sparing surgery, the breast was irradiated with tangential fields at the dose of 50 Gy in 25 sessions, plus a 10 Gy in 5 sessions boost to the scar. After radical mastectomy, 40 Gy in 20 sessions were delivered to the tumour area in cases with of pT4 disease. The axillary region was irradiated if more than 3 lymph nodes were involved at histological examination.

The RECIST criteria for response evaluation were used^16^, while toxicity was classified according to the Common Toxicity Criteria of the National Cancer Institute.^17^ According to RECIST criteria, a 30% reduction in the longest diameter of the target lesion was required to qualify it for partial response. The pathological complete response (pCR) was defined as the absence of infiltrating and/or in situ carcinoma in the surgical specimen (breast and lymph nodes). Overall survival and progression free survival were calculated from the start of therapy until death or progression of disease, respectively. Progression free survival and overall survival curves were plotted by the Kaplan-Meier method.^18^

From March 2002 to May 2006, 13 Series I patients and from May 2006 to May 2010, 37 Series II patients were entered into the studies. The patient characteristics are reported in Table 1.

## Results

### Series I patients (n=13)

Out of the 13 patients, 12 received the planned 4 cycle of preoperative chemotherapy. Surgery was anticipated in one case due to the patient’s preference. No patient progressed during the preoperative phase.

Clinical responses, evaluated before planned surgery, included complete response in 1 patient, partial response in 10, stable disease in 1, progression in 1. Response rate (responding/entered patients) was 84% (95% confidence limits, 64–100%). All 13 patients underwent surgery (radical mastectomy in 10, quadrantectomy in 3). The histological examination of the breast revealed no signs of disease in 3 patients, infiltrating carcinoma in 10. Pathological T classification was T0 in 3 patients, T1b in 1, T1c in 2, T2 in 3, T3 in 1, T4 in 3. In 2 patients with small residual primary tumour the disease was multifocal, precluding conservative surgery. Pathological tumour downstaging occurred in 3/4 T2, 4/5 T3, 3/4 T4 cases. The median number of examined axillary lymph nodes was 23 (range 1–29). In 1 patient less than 5 lymph nodes could be identified in spite of meticulous search. Lymph nodes were pathologically negative in 2 patients, positive in 11 patients. The median number of positive lymph nodes was 6 (range 2–22). Eight patients had more than 3 lymph nodes involved. A pCR (no evidence of disease both in the breast and in the axilla) was achieved in 7.6% (1/13) of patients. All 13 patients started with postoperative chemotherapy as planned and 8 of them received all 4 planned cycles (8 cycles altogether). One patient did not receive postoperative epirubicin and taxotere because of poor clinical/pathological response to neoadjuvant treatment. This patient received CMF postoperatively.

Twelve patients received adjuvant hormonal therapy and 9 underwent radiation therapy. Six events (5 distant metastases, 1 loco-regional relapse and 4 deaths, all due to progressive disease) occurred at a median follow up of 44.1 months.

### Series II patients (n=37)

All patients completed the 4 pre-evaluation cycles. Anticipated surgery was offered to 3 patients due to lack of response and in 2 due to the patient’s preference. Thirty-two patients were able to complete the 8 cycle preoperative phase. No patient progressed during preoperative chemotherapy.

Clinical responses, evaluated before planned surgery, included complete remission in 13 patients, partial remission in 21, stable disease in 3. Response rate (responding/entered patients) was 92% (95% confidence limits, 84–100%). All 37 patients underwent surgery (radical mastectomy in 22, quadrantectomy in 15). The histological examination of the breast revealed no signs of disease in 8 patients, infiltrating carcinoma in 29. Pathological T classification was T0 in 8 patients, T1a in 6, T1b in 4, T1c in 11, T2 in 5, T3 in 2, T4 in 1. In 1 patient with small residual primary tumour the disease was multifocal, precluding conservative surgery. Pathological tumour downstaging occurred in 21/24 T2, 10/10 T3, 2/3 T4 cases. The median number of examined axillary lymph nodes was 14 (range 1–32). Lymph nodes were negative in 15 patients, positive in 22 patients. The median number of positive lymph nodes was 3 (range 1–10). Ten patients had more than 3 lymph nodes involved. A pCR (no evidence of disease both in the breast and in the axilla) occurred in 10.8% (4/37) of patients. Thirty-four patients received postoperative CMF for 3 cycles as planned, 3 declined further chemotherapy.

Thirty-six patients received adjuvant hormonal therapy and 25 underwent radiation therapy. Two events (1 distant metastasis, 1 loco-regional relapse and no deaths) occurred at a median follow up of 37.5 months.

**FIGURE 1.**
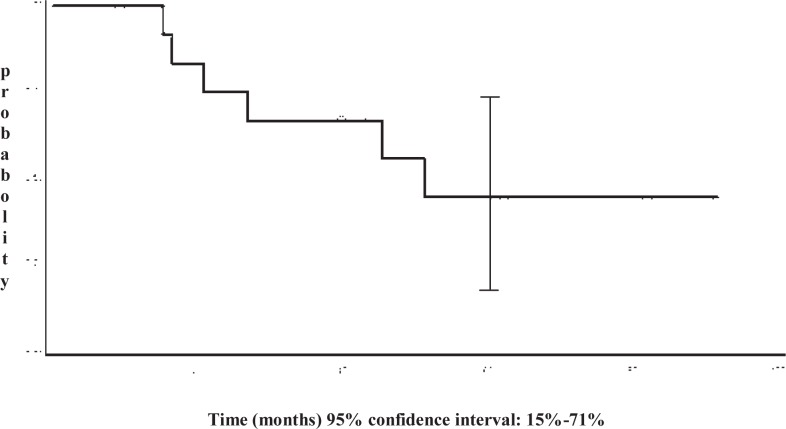
Progression free survival of 13 patients of the Series I.

Altogether, only one of 9 patients with a lobular histology and none of 14 patients with a low proliferation index (Ki-67 < 14%) achieved a pCR. The toxicity encountered in the neoadjuvant phase in the 2 patient series is reported in Table 2. Overall, toxicity was acceptable and no toxic deaths occurred. Progression free survival curves are presented in Figure 1. In Series I, median progression free survival was 51 months and 5-year progression free survival was 44% (95% confidence interval: 15%- 71%). Median overall survival has not been reached yet.

In Series II, median progression free survival has not been reached yet and 5-year progression free survival was 93% (95% confidence intervals, 75%– 98%).

## Discussion

Several studies have reported high clinical response rates following preoperative chemotherapy, but the pCR rates have remained relatively low. The results of studies large enough to permit a subtype analysis have shown that pCRs tend to concentrate in patients with HER2 overexpressing or triple negative tumours.^12^–^14^ Moreover, lobular carcinoma has been associated with poor response to chemotherapy.^19^

In addition, an early response to and the duration of chemotherapy has been shown to increase the pCR rate. For instance, the combination of docetaxel, Doxorubicin and Cyclophosphamide for 2 cycles followed by either 4 additional cycles of the same regimen or by 4 cycles of vinorelbine and capecitabine yielded a 7.3 and 3.1% respectively pCR rate in patients who did not respond clinically to the first 2 chemotherapy cycles, while in initially responding patients the final pCR rate was 22.6%.^7^ In the large NSABP B-27 trial, it was shown that the addition of 4 docetaxel cycles after 4 cycles of Doxorubicin plus Cyclophosphamide alone increased the pCR rate from 13.7% to 26.1%.^20^

In a previous series of 45 evaluable patients treated with 4 cycles of neoadjuvant ET at our Institution, 7 (16%) showed no signs of disease and 2 additional patients presented only carcinoma in situ at histological examination of the breast 15. However, some of these patients had persistent nodal disease, leaving only 3 patients with pCR. Although numbers of patients were small, pCR occurr more often in ER negative (9%) than in ER positive patients (4%). These unsatisfactory results might have been related to the limited number of preoperative chemotherapy courses (only 4 cycles were administered). We added, therefore, in a subsequent study 4 additional chemotherapy cycles in the preoperative setting and limited inclusion criteria to hormonal receptor positive, HER2 negative cases.

With the aim to gain insight in the niche of ER positive, HER-2 negative cases, we report on the updated outcome of cases treated with 4 chemotherapy cycles and on the outcome of patients included in the subsequent study, treated with 8 cycles of neoadjuvant chemotherapy. The present study adds to the body of evidence showing that preoperative chemotherapy of breast cancer is of limited value in hormone receptor positive, HER2 negative cases. No obvious increase in the pCR rate by increasing the number of chemotherapy cycles was achieved (10.8% *vs*. 7.6%), but numbers are too small for comparison and differences in the distribution of prognostic factors, starting from tumour size, in the two series of patients should be taken into account.

The impact of other parameters possibly influencing pCR rate in these patients, i.e. proliferation index and histological type was in agreement with the reported chemoresistance of tumours with lobular histology and/or low proliferation rate.^12^,^19^ the progression free survival was longer (90% *vs*. 38% at 5 years) in patients treated with 8 preoperative cycles than in those receiving 4 only, but again the nature of the study precludes comparisons.

Based on the analysis of large, randomised studies^14^, once a decision is taken, based on several considerations such as patient’s age, histology, tumour grade and proliferation, to offer an ER positive, HER2 negative patient preoperative chemotherapy, the latter should be given for a sufficient period of time, probably in the order of 6 months.^3^ Our findings reassure us on the benefit of a longer chemotherapy duration with the inherent toxicities. The hormonal therapy has emerged as a viable alternative to chemotherapy in patients with a little chance of chemotherapy response, particularly in postmenopausal women.^21^

The continuing evaluation of biologic features should permit a treatment tailoring aimed to offer cytotoxic chemotherapy only to patients who have a substantial chance of deriving benefit from it.

## Figures and Tables

**TABLE 1.** Patient characteristics

	**SERIES I**	**SERIES II**
Total number of patients	13	37
Median age (range)	46 years (23–65)	46 years (27–67)
Menopausal status		
Premenopausal	7	28
Postmenopausal	6	9
Stage: T2	4	24
T3	5	10
T4	4	3
Inflammatory signs	3	1
N0/N1	9/13	15/22
Histology		
Ductal	12	29
Lobular	1	8
Hormone receptor status		
ER+/PgR+	9	23
ER+/PgR-	3	14
ER-/PgR +	1	0
Ki-67 (%)		
< 14	-	14
> 14	-	9
Unknown	13	14

**TABLE 2.** Toxicity of chemotherapy (Series I *vs*. Series II)

**Toxicity**	**All grades (%)**	**G1 (%)**	**G2 (%)**	**G3 (%)**	**G4 (%)**
**Neutropenia**	12 (92) vs 37 (100)	0 vs 0	1 (7) vs 0	1 (7) vs 2 (5)	10 (77) vs 35 (95)
**Thrombocytopenia**	0 vs 0	0 vs 0	0 vs 0	0 vs 0	0 vs 0
**Anaemia**	6 (46) vs 19 (51)	5 (38) vs 11 (29)	1 (7) vs 7 (18)	0 vs 1 (2)	0 vs 0
**Nausea and vomiting**	7 (53) vs 13 (35	3 (23) vs 2 (5)	4 (30) vs 11 (29)	0 vs 0	0 vs 0
**Mucositis**	8 (61) vs 20 (54)	4 (30) vs 9 (24)	3 (23) vs 7 (18)	1 (7) vs 3 (8)	0 vs 1 (2)
**Diarrhoea**	2 (15) vs 4 (10)	2 (15) vs 1 (2)	0 vs 3 (8)	0 vs 0	0 v s0
**Onicopathy**	1 (7) vs 5 (13)	0 vs 2 (5)	1 (7) vs 2 (5)	0 vs 0	0 vs 0
**Asthenia**	7 (53) vs 18 (48)	6 (46) vs 7 (18)	1 (7) vs 11 (29)	0 vs 0	0 vs 0
